# Development of a trunk motor paradigm for use in neuroimaging

**DOI:** 10.1515/tnsci-2020-0116

**Published:** 2020-06-01

**Authors:** Elizabeth Saunders, Brian C. Clark, Leatha A. Clark, Dustin R. Grooms

**Affiliations:** Division of Athletic Training, School of Applied Health Sciences and Wellness, College of Health Sciences and Professions, Ohio University, Athens, OH, 45701, United States of America; Physical Therapy and Sports Medicine Centers, New London, CT, 06320, United States of America; Ohio Musculoskeletal and Neurological Institute, Ohio University, Athens, OH, 45701, United States of America; Department of Biomedical Sciences, Ohio University, Athens, OH, 45701, United States of America; Ohio Musculoskeletal and Neurological Institute, Ohio University, Athens, OH, 45701, United States of America; Department of Family Medicine, Ohio University, Athens, OH, 45701, United States of America; Ohio Musculoskeletal and Neurological Institute, Ohio University, Athens, OH, 45701, United States of America; Division of Athletic Training, School of Applied Health Sciences and Wellness, College of Health Sciences and Professions, Ohio University, Athens, OH, 45701, United States of America

**Keywords:** low back pain, motor control, motor paradigm, neuroimaging, fMRI

## Abstract

The purpose of this study was to quantify head motion between isometric erector spinae (ES) contraction strategies, paradigms, and intensities in the development of a neuroimaging protocol for the study of neural activity associated with trunk motor control in individuals with low back pain. Ten healthy participants completed two contraction strategies; (1) a supine upper spine (US) press and (2) a supine lower extremity (LE) press. Each contraction strategy was performed at electromyographic (EMG) contraction intensities of 30, 40, 50, and 60% of an individually determined maximum voluntary contraction (MVC) (±10% range for each respective intensity) with real-time, EMG biofeedback. A cyclic contraction paradigm was performed at 30% of MVC with US and LE contraction strategies. Inertial measurement units (IMUs) quantified head motion to determine the viability of each paradigm for neuroimaging. US vs LE hold contractions induced no differences in head motion. Hold contractions elicited significantly less head motion relative to cyclic contractions. Contraction intensity increased head motion in a linear fashion with 30% MVC having the least head motion and 60% the highest. The LE hold contraction strategy, below 50% MVC, was found to be the most viable trunk motor control neuroimaging paradigm.

## 1 Introduction

Low back pain (LBP) is a common health concern afflicting a large portion of the population [1,2,3]. Cross-sectional studies estimate that 15–30% of the general population is experiencing LBP at any one time [1,2,3,4]. Similarly, an estimated 60–80% of the general population is expected to experience LBP throughout their lifetime [1]. While many who suffer LBP experience relief within a short amount of time (i.e., 1–4 weeks), many experience recurrent episodes of pain [3]. The pervasive nature of LBP results in medical, financial, and quality of life burdens, making research into the mechanisms of LBP and possible therapeutic initiatives a national healthcare priority [1,5,6].

Individuals living with chronic LBP often suffer motor control deficits in trunk musculature [4,7,8]. For instance, Hodges and Richardson identified significant delays in the transverse abdominis (TA) and lumbar erector spinae (ES) muscle activation among individuals with LBP [4,7]. Similar studies have purported postural control impairments among individuals with LBP [8]. Radebold et al. assessed balance performance in unstable sitting conditions and during a trunk muscle force release task among individuals with chronic LBP [8]. Those with LBP had delayed muscle response times and decreased ability to regulate force, as well as poorer balance control relative to matched healthy participants [8]. These findings suggest a connection between LBP and depressed neural control of the trunk musculature [4,8,9].

Recent work has provided compelling evidence of altered cortical activity in those with chronic LBP to painful stimuli [4,10,11]. Kobayashi et al. studied the effects of mechanically stimulated LBP on cortical activity and self-reported pain sensitivity via functional magnetic resonance imaging (fMRI). The mechanically stimulated pain was induced via the application of three, 30 s blocks of standardized pinpoint posterior-to-anterior manual pressure to induce pain between a three and five on the visual analog scale, at the fourth–fifth lumbar spinal interspace [10]. Results identified increased activity in the insular, supplementary motor, secondary somatosensory, and posterior cingulate cortexes, as well as significant hyperalgesia among individuals with chronic LBP as compared with healthy controls [10]. These findings provide insight into the pathomechanics of chronic back pain; however, there has been limited neuroimaging investigation into trunk motor function specifically. While success has been achieved to quantify the motor representation with transcranial magnetic stimulation [12,13], the neural activity to generate voluntary trunk muscle activity has yet to be quantified. In fact, to our knowledge, neuroimaging of trunk motor function is limited to one case study examining the effects of pain physiology education on brain activity during an abdominal draw-in maneuver [14].

The development of a viable trunk motor neuroimaging paradigm has the potential to yield unparalleled insight into the relationships between LBP, motor control of the trunk muscles, and altered neural activity. Currently, no validated trunk motor paradigms exist that may be used in an fMRI setting. Typical fMRI motor paradigms for the upper or lower extremity tend to use frequent, small amplitude motions in blocked cycled contractions to generate a robust blood-oxygen-level-dependent (BOLD) response [15,16,17,18,19,20]. However, this method may not be suitable for a trunk motor paradigm due to potential increases in head motion when engaging axial skeletal musculature. Due to the anatomical nature of the trunk muscles, any pelvic or spinal motion is likely to induce translations up the spine and thus the head, which has prevented the implementation of trunk muscle neuroimaging motor paradigms. Our long-term goal is to develop a viable neuroimaging trunk muscle motor neuroimaging technique. As a first step toward this goal, in this article, we report our results comparing head motion across various contraction strategies, intensities, and paradigms.

## 2 Materials and methods

### 2.1 General overview of the experimental design

The primary objective of this study was to examine the magnitude of head motion between contraction strategies (isometric upper spine vs isometric lower extremity) across four contraction intensities (30% of maximal voluntary contraction (MVC), 40% MVC, 50% MVC, and 60% MVC). All MVC and relative muscle contraction intensity data were based on the amplitude of the electromyogram signal. EMG was selected as it has potential utility to normalize contraction intensity in the MRI environment [21,22,23,24,25]. A secondary objective was to compare head motion during two different contraction paradigms (cyclic contractions vs static hold contractions). Cyclic contractions may tend to increase BOLD response to a greater degree than hold contractions [26,27,28,29] but may induce more head motion due to the repeated contractions. Cyclic contractions were only completed at 30% MVC after pilot testing demonstrated excessive head motion (i.e., >0.5 mm) with isometric contractions above this intensity level when utilizing either contraction strategy. Conversely, hold contractions demonstrated the potential to keep head motion to a minimum during the pilot testing; therefore, this paradigm was tested at all contraction intensities. This required participants to complete 10 sets of different erector spinae (ES) contractions. Each set consisted of four 15 s blocks of contraction interspersed with five 15 s blocks of rest. The order of intensity and contraction strategy was counterbalanced among participants.


**Ethical approval:** The research related to human use has been complied with all the relevant national regulations, institutional policies and in accordance the tenets of the Helsinki declaration and has been approved by the authors’ institutional review board or equivalent committee.
**Informed consent:** Informed consent has been obtained from all individuals included in this study.

### 2.2 Participants

Ten healthy volunteers participated in the study (four male, six female; 22.9 ± 1.1 years; 72.6 ± 18.6 kg; 1.7 ± 0.1 m). Participants completed informed consent documentation and were excluded if they reported any neurological and musculoskeletal impairments (Health History Questionnaire), including a history of chronic low back pain as determined by the Health Assessment Questionnaire (HAQ) and Oswestry Low Back Disability Inventory. We chose to conduct our initial development experiments in healthy individuals (as opposed to those with low back pain) as we were concerned that the large number of contractions required to complete the experiment could exacerbate pain and/or be difficult for some individuals with low back pain to complete.

### 2.3 Instrumentation, electromyography, overview of paradigm

Noraxon inertial measurement units (IMUs) quantified degrees of head course, pitch, and roll associated with contraction paradigms. IMUs were placed along the superior borders of participants’ frontal bone of the skull and sternum.

Electromyographic (EMG) signals were recorded using the TeleMyo DTS System (Noraxon, Scottsdale, AZ) bilaterally from the belly of the ES muscles at the third–fourth lumbar vertebrae using a bipolar electrode configuration (Ag/Ag Cl Noraxon dual-electrodes (part 272) with a 2.5-cm center-to-center interelectrode distance) [30]. A reference electrode was located on the bony aspect of the left anterior superior iliac crest. Prior to applying the electrodes, the skin was shaved, cleaned with alcohol, and abraded to minimize impedance. EMG recordings were made using pre-amplified (500×) wireless sensors with a common-mode rejection ratio > 100 dB (Model 548 DTS Lossless EMG sensor, Noraxon, Scottsdale, AZ) (preamplifiers were located on the lateral aspect of the trunk/hip). These signals were bandpass filtered (10–500 Hz) and sampled at a rate of 1,000 Hz using a 16-bit ADC (Noraxon USA) and software (myoRESEARCH-XP software, Noraxon USA). The EMG signals were subsequently ECG reduced and full-wave rectified, and the peak root-mean-squared (RMS) EMG activity over a 150 ms window was calculated. For the relative intensity contraction where biofeedback (more details later) was provided, the RMS EMG was normalized to the highest RMS EMG calculated from three MVCs (more details later) [31].

Participants then laid supine on a treatment table and performed three practice sets of the upper spine (US) and lower extremity (LE) contractions, as described later. Next, participants performed the tasks at the respective contraction intensity for each contraction strategy (US and LE). To determine the contraction intensities, participants performed three isometric MVCs for each contraction strategy with 2 min rest between each. To complete the US press MVC, participants were instructed to “use your back muscles to isometrically press your shoulder blades into [the researcher’s] hands as hard as possible”. The researcher gave feedback and monitored for the isolated isometric spinal extension with minimal scapular retraction, cervical extension, or other accessory motions. To complete the MVC for the lower extremity (LE) press, participants were instructed to “use your back muscles to isometrically press your heels into [the researcher’s] hands as hard as possible”. The researcher gave feedback to ensure isolated isometric spinal extension with minimal knee flexion, hip extension, vertebral arching, or other accessory motions.

### 2.4 Data collection

**Figure 1:**
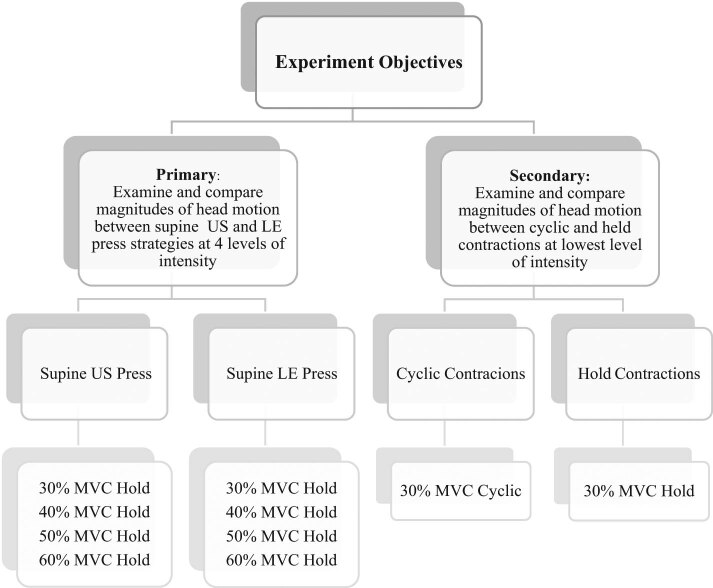
Experimental objectives and design. MVC: maximum voluntary contraction; US: upper spine; LE: lower extremity.

US and LE contraction strategies were performed using a block design with 15 s of rest alternating with 15 s of cued voluntary isometric contraction for four blocks. Each contraction block was performed with EMG biofeedback for targeted intensities of ES contraction (Figure 1). Trial order was counterbalanced between participants to account for learning or fatigue effects. Real-time auditory feedback was provided through Noraxon MyoMotion software to facilitate instruction on contraction intensity performance. When participants exerted ES force higher than specified parameters (i.e., 10% MVC above the target level), Noraxon auditory cues instructed individuals to “reduce the activity”. Similarly, when participants exerted ES force less than specified parameters (i.e., 10% MVC below the target level), Noraxon auditory cues instructed individuals to “increase the activity”. Participants practiced each contraction intensity 3–5 times with auditory feedback, followed by 1–3 times without auditory feedback. Recorded trials consisted of one contraction repetition with feedback and 3 repetitions without feedback to simulate one potential application in the fMRI environment (initial practice session before scanning and only start-stop feedback during scanning).

### 2.5 Statistical analysis

Head pitch (sagittal plane rotation) was used as the primary dependent variable because it was the only range of motion that approached levels of sufficient head artifact to impact data quality (>3° or approximately 0.5 mm [32,33] of translation with an IMU depth of 1 cm). Two separate two-way repeated-measures ANOVAs (RM-ANOVAs) were performed. The dependent variable in both of these analyses was head pitch. In the first RM-ANOVA, the independent variables were contraction intensity (4 levels: 30%, 40%, 50%, 60% MVC) and contraction strategy (2 levels: LE and US). In the second RM-ANOVA, the independent variables were contraction paradigm (2 levels: hold and cyclic) and contraction strategy (US and LE). A Sidak *post hoc* test was used to determine differences when the main effect and/or interaction term was significant. A pre-set alpha level of significance was set at *p* < 0.05 for all analyses. We report eta-squared (*η* ^2^) to aid in data interpretation. To determine practicality for use as a neuroimaging paradigm, we also report descriptive data for the percentage of participants and percentage of blocks across each contraction strategy, paradigm, and intensity level that would be considered potentially viable for neuroimaging (<0.5 mm of head translation) [32].

## 3 Results

### 3.1 Analysis 1: Effect of contraction intensity and contraction strategy

There was no contraction intensity (30%, 40%, 50%, 60%) × contraction strategy (US vs LE) interaction (*p* = 0.76; *η* ^2^ = 0.02). Additionally, there was no main effect for contraction strategy (*p* = 0.18; *η* ^2^ = 0.07). There was, however, a main effect for contraction intensity (*p* < 0.01; *η* ^2^ = 0.48); Table 1). Here, *post hoc* tests with Sidak correction indicated that 30% MVC had significantly lower head pitch than 60% MVC (*p* = 0.049) with no other contraction intensity differences.

**Table 1:** Head pitch ROM between contraction intensities and contraction strategies for hold contraction paradigm

	US head pitch ROM	LE head pitch ROM
30% MVC	1.39 ± 0.62*	1.29 ± 0.59*
40% MVC	1.55 ± 0.57	1.39 ± 0.44
50% MVC	1.77 ± 0.82	1.73 ± 0.79
60% MVC	2.10 ± 0.92*	1.88 ± 1.01*

Head motion across contraction intensity (30–60% MVC) and contraction strategy (upper spine and lower extremity). No differences by contraction strategy, *indicates significant difference for contraction intensity (30% MVC relative to 60% MVC, *p* < 0.05 Sidak corrected); mean ± standard deviation reported.

### 3.2 Analysis 2: Effect of contraction paradigm and contraction strategy

**Figure 2:**
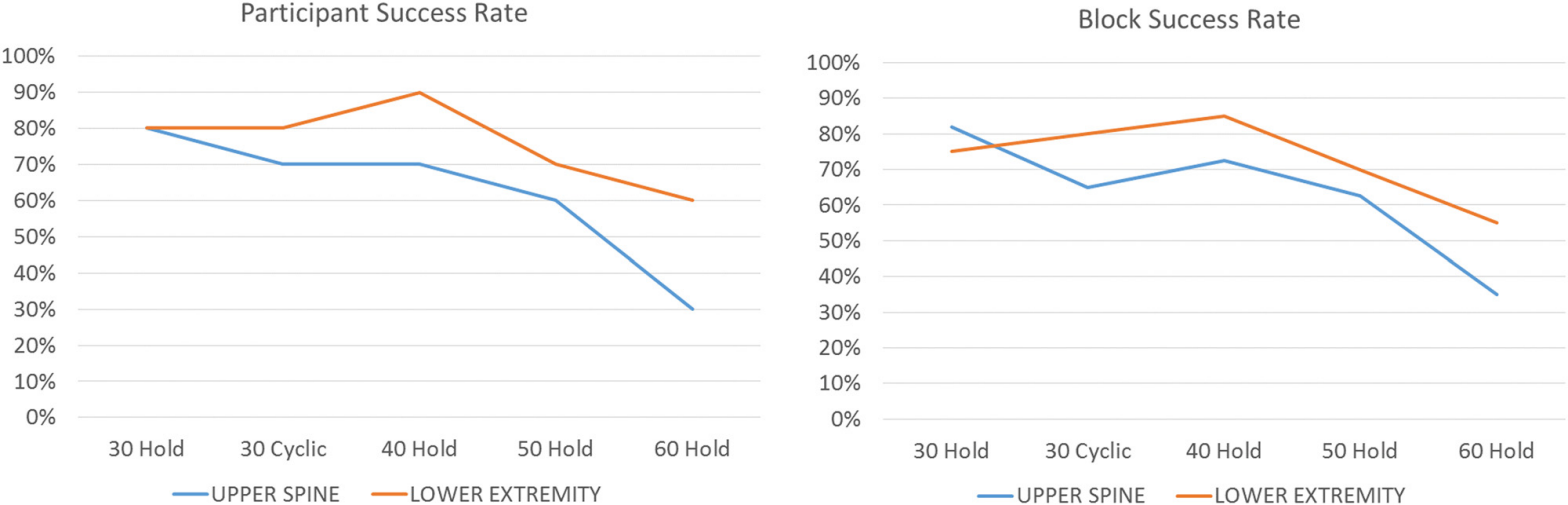
Block and participant success rate between contraction strategy and paradigm. (left) Percentage of successful (acceptable head motion) participants under each contraction paradigm, strategy, and intensity level and (right) percentage of successful blocks across all subjects that did not have excessive head motion.

There was no contraction paradigm (hold vs cyclic) × contraction strategy (US vs LE) interaction (*p* = 0.97; *η* ^2^ < 0.00). There was also no main effect for contraction strategy (*p* = 0.25; *η* ^2^ = 0.08). There was, however, a main effect for contraction paradigm (*p* = 0.01; *η* ^2^ = 0.32); Table 2. Here, the “hold” paradigm elicited 14% less head pitch relative to “cyclic” paradigm. The LE contraction strategy had over 70% success for less than 0.5 mm head motion with contraction intensities below 50%, whereas the UE contraction strategy was only 70% successful through 40% contraction intensity (Figure 2).

**Table 2:** Head pitch ROM between contraction strategies for hold and cyclic contraction paradigms

	US head pitch ROM	LE head pitch ROM
30% MVC hold	1.39 ± 1.27*	1.29 ± 1.14*
30% MVC cyclic	1.59 ± 1.34	1.50 ± 1.48

Head motion during cyclic and hold contraction paradigms across the upper spine and lower extremity contraction strategies. *indicates *p* < 0.05 for the difference in head motion between hold and cyclic contractions; mean ± standard deviation reported.

## 4 Discussion

Our long-term goal is to develop a viable neuroimaging trunk muscle motor neuroimaging technique. Herein, we report our results comparing head motion across various contraction strategies, intensities, and paradigms. Our acceptable criteria for the head motion was under 3° rotation (approximately 0.5 mm translation) in any direction selected *a priori* as a common neuroimaging threshold for data quality check [32]. Head pitch was the only motion approaching this excessive threshold (>3°); therefore, we focused on head pitch during each motor block and determined the percentage of successful trials (15 s block without head motion above 3°) and percentage of subjects with all successful trials. Our findings revealed that both US and LE contraction strategies are valid methods to assay trunk motor function during neuroimaging with minimal head movement. Sustained trunk contractions yielded less head motion as compared with repeated trunk contractions, and isometric trunk contractions are viable when performed below 50% of MVC, as increases in contraction intensity concurrently increase head motion beyond acceptable limits. Below, we further discuss these findings.

### 4.1 Use of novel trunk motor paradigm for quantifying brain activity

Increases in head motion during cyclic trunk contractions relative to hold trunk contractions indicate that the typical cyclic motor paradigm, most commonly used in fMRI motor control research due to subsequent increases in the BOLD signal, may not be ideal for the trunk muscles [15,16,17,18,19,20,34]. Cyclic trunk contractions elicited significantly more head motion, even when performed at low levels of ES muscle activity. Cyclic contractions of trunk musculature may be difficult to reproduce in a neuroimaging setting due to the involvement of multi-articular muscles across the vertebral column, increasing motion translation to the head as opposed to investigations that quantify appendicular skeletal motion [15].

While there were no significant differences in head motion between LE and US contraction strategies, the LE press technique typically resulted in increased rates of participant and block success, ranging from 10% to 30% above the US contraction strategy. Thus, while statistically, head motion was not different, this increased probability of data collection success with head motion below 0.5 mm indicates the LE strategy may be preferred in fMRI settings as minimal head motion is typically preferred [15,32]. Increased contraction intensity induced significant increases in head motion, with contraction levels below 60% of participants’ MVC yielding a higher probability of block or overall participant success with minimal head motion (<3°). Utilizing trunk contractions above 50% of an individual’s MVC may not be suitable for use in fMRI settings due to associated increases in head motion [15,32].

Similar research assaying the efficacy of novel fMRI paradigms has minimized head motion by means of external fixation of the head and/or spine, joint angle fixation, and implementing motor tasks performed at slow speeds [16,35,36,37]. Newton et al. successfully limited head movement during a lower extremity motor task by fixating flexed hip and knee joints (45° flexion) to a mounted polyethylene wedge [15]. This posture allows rotational forces to occur primarily at the lower limb rather than propagating through the spine to the head [15]. This form of fixation is not reproducible for an MRI-compatible trunk motor paradigm due to spinal mechanics and anatomical consideration of restraining vertebral segments that may disrupt the ES muscle contractions. Multiple studies evaluating novel fMRI paradigms utilized external fixations of the head and spine to limit head motion artifact [16,35,36,37]. Forms of external fixation used in similar studies included mounted straps at the head, shoulders or hips, vacuum pillows placed under participants’ backs, and inflatable cushions positioned under participants’ heads and shoulders [16,35,36]. While these methods are useful in minimizing head motion associated with peripheral motor tasks, these fixation devices would likely diminish an individual’s ability to perform trunk motor tasks, as many involve restraint across the torso. Therefore, this investigation did not utilize immobilization techniques or the typical extensive padding or restraints of fMRI motor paradigms to ensure adequate trunk muscle activity. When translating these results to the MRI, additional padding around the head may further reduce movement artifact, but we advise caution with trunk restraint as that may affect trunk motor performance, and we achieved reasonably low head motion with a hold LE contraction strategy under 50% MVC.

### 4.2 Implications

This study has provided basic evidence toward the development of a suitable trunk motor protocol for use in neuroimaging settings. We believe these findings can be used to refine a protocol that can be successfully (e.g., reliably) translated into the MRI environment. If we are ultimately successful in this translation, these findings will allow for further research in a complementary fashion with established paradigms employing electroencephalography, EMG, transcranial magnetic stimulation, and resting state or structural brain studies, into the examination of neural adaptation associated with trunk muscle function and the exploration of neural mechanisms associated with LBP. Current evidence purports motor cortex reorganization associated with recurrent LBP [4]. Reduced motor thresholds were identified among individuals with LBP using transcranial magnetic stimulation, as well as posterior-lateral shift in the center of gravity for peak motor evoked potential location [4]. These findings indicate possible changes in cortical organization associated with activation of trunk musculature; however, these results are limited by the external nature of the simulation and are representative of voluntary trunk motor function [4]. While fMRI technology has been used to identify cortical changes regarding pain sensitivity among individuals with chronic LBP [10], far more limited neuroimaging has been completed to quantify trunk motor functionality. The lack of assessing trunk motor function during neuroimaging has limited inferences on the neural interactions of pain stimuli, motor control adaptations, and cortical reorganization. This work will allow future research utilizing fMRI to assess variances in cortical activation patterns during trunk contractions in the evaluation of neural mechanisms associated with LBP.

### 4.3 Limitations

These data were derived from a small sample of healthy participants. Our results suggest that head motion artifact occurs within the limits of accepted standards (<0.5 mm or <3°) while performing sustained trunk muscle contractions below 50% MVC [15,32]. However, these findings may not be transferable to samples with low back pain or other motor control deficits that may decrease capability to contract the trunk muscles. Furthermore, motor tasks utilized in this research were not performed in a magnetic resonance (MR) scanner or simulator. Tasks performed in an MR setting may provoke anxiety among participants and elicit higher degrees of head motion. Also, the use of EMG to normalize the trunk motor contractions was selected as it is a viable tool to be used during fMRI and provides a means to capture muscle contraction activity in an isometric fashion with minimal disturbance to the supine position requirements of fMRI. However, it should be noted that extrapolations to relative force production from the EMG data is not viable (i.e., the EMG-force relationship is not linear) [38,39,40,41]. Lastly, the nature of eliciting isolated paraspinal muscle contractions with minimal accessory motion may result in other muscles also activating such as hip extensors or knee flexors.

## 5 Conclusion

Both US and LE contraction strategies are valid methods to assay trunk motor function during neuroimaging with minimal head movement. Sustained trunk contractions yield significantly less head motion as compared with repeated trunk contractions. Isometric trunk contractions are viable when performed below 50% of MVC, as increases in contraction intensity concurrently increase head motion beyond acceptable limits. Therefore, the lower extremity press contraction strategy, below 50% MVC completed in an isometric sustained fashion maybe a viable trunk motor control neuroimaging paradigm.
